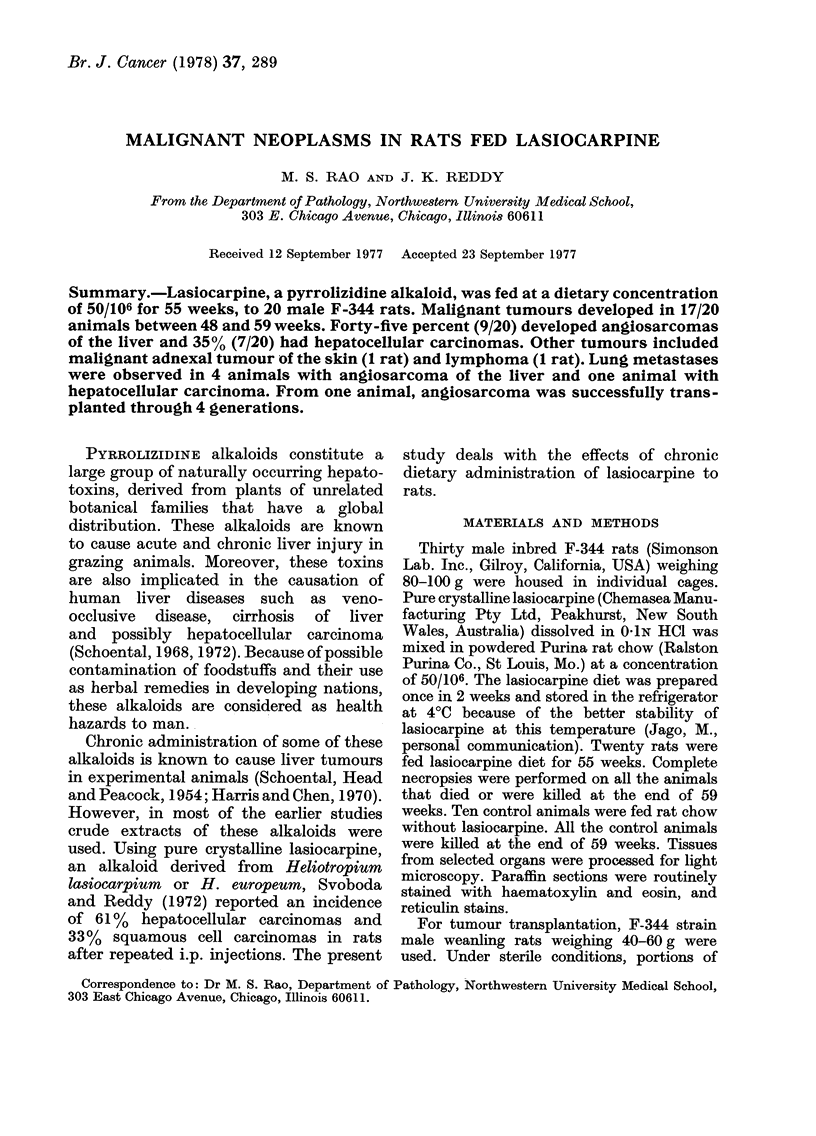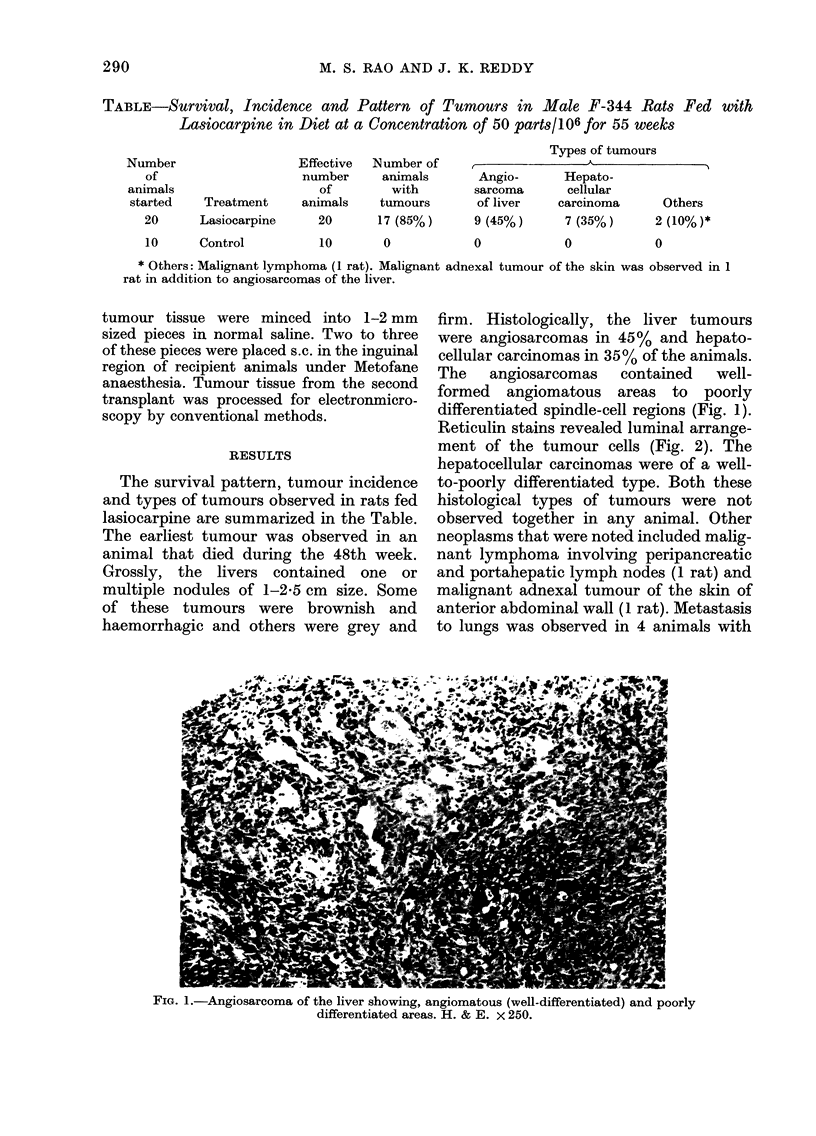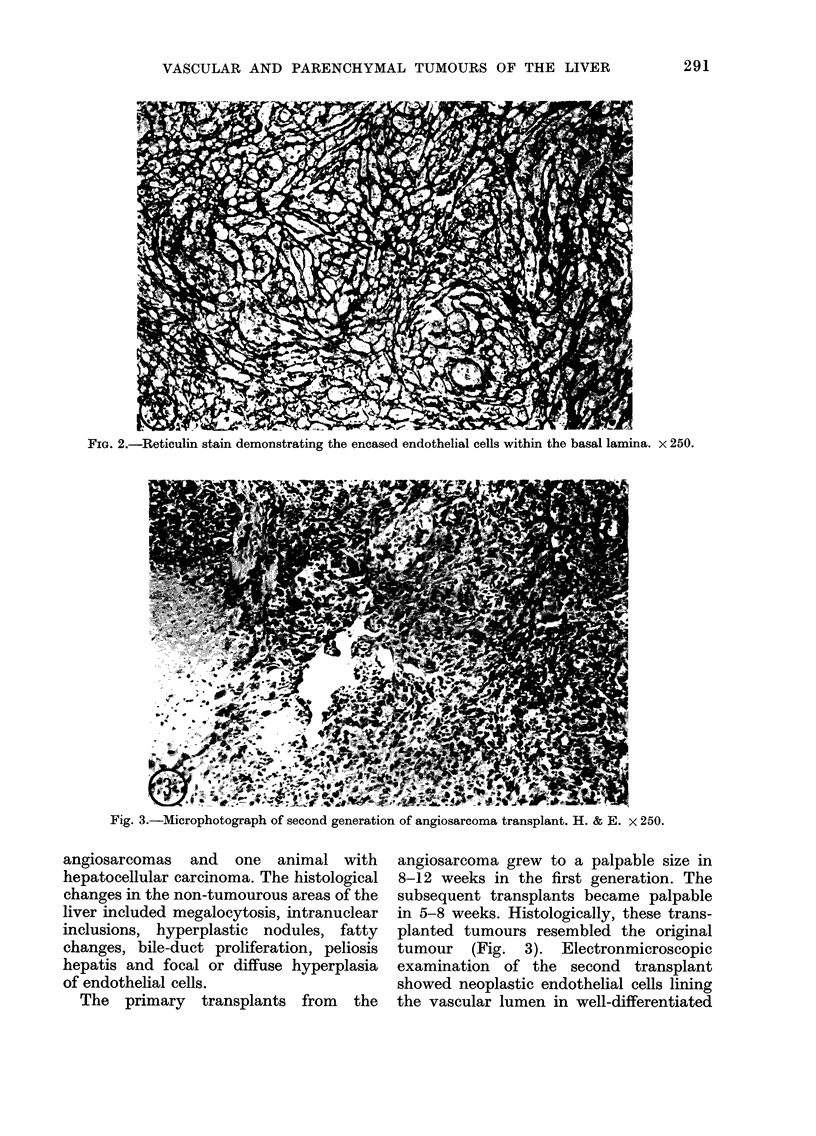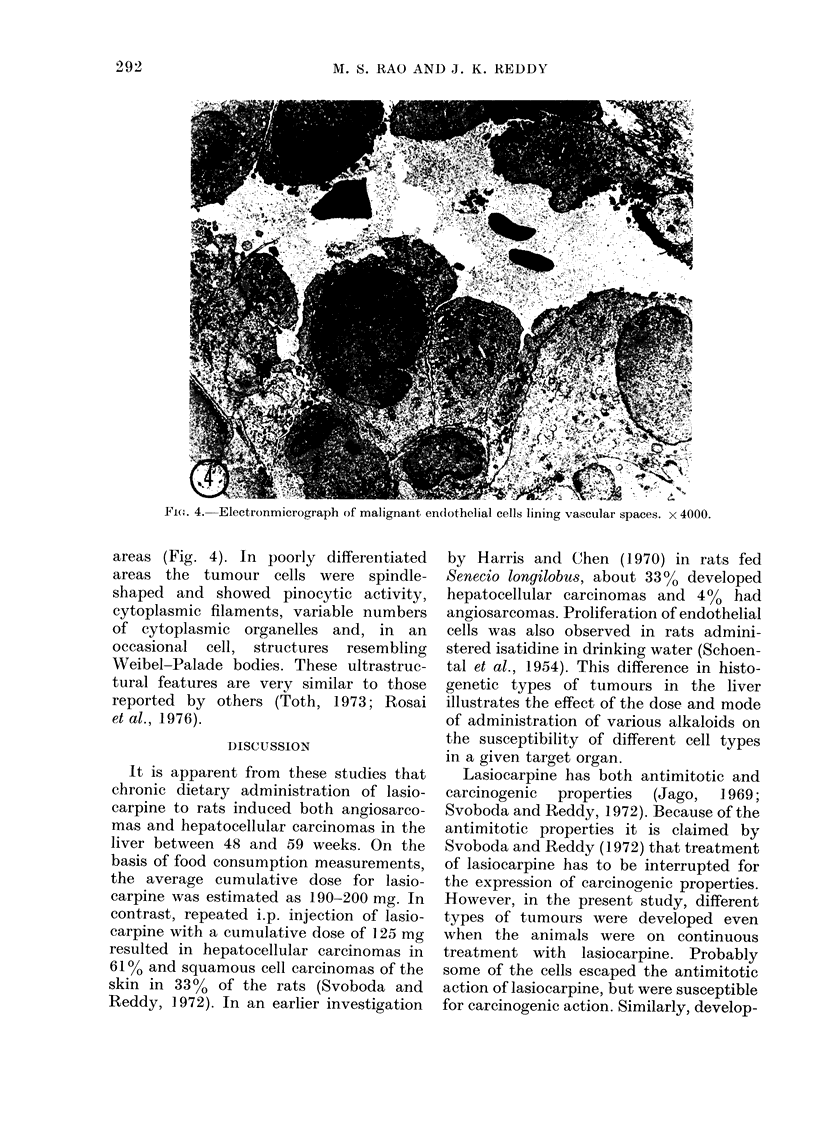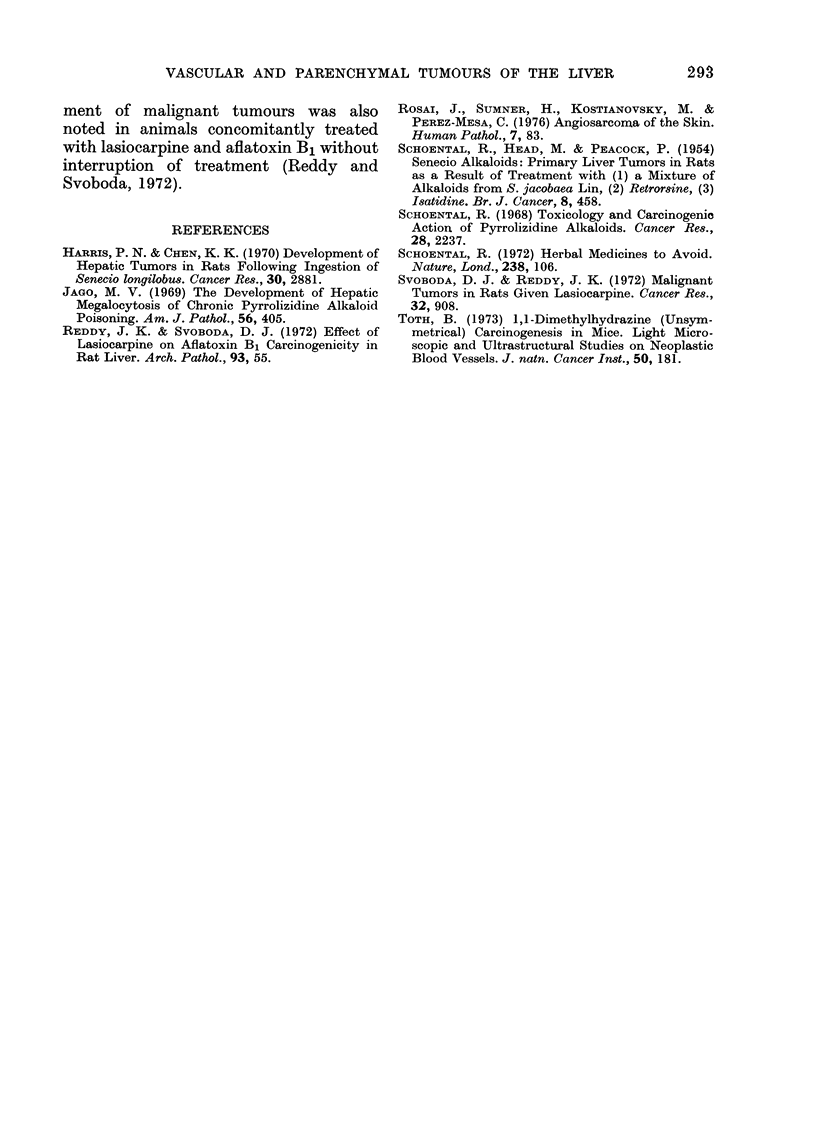# Malignant neoplasms in rats fed lasiocarpine.

**DOI:** 10.1038/bjc.1978.38

**Published:** 1978-02

**Authors:** M. S. Rao, J. K. Reddy

## Abstract

**Images:**


*Br. J. Cancer* (1978) **37**, 289

### MALIGNANT NEOPLASMS IN RATS FED LASIOCARPINE

M. S. RAO AND J. K. REDDY

*From the Department of Pathology, Northwestern University Medical School, 303 E. Chicago Avenue, Chicago, Illinois* 60611

Received 12 September 1977 Accepted 23 September 1977

**Summary.—Lasiocarpine, a pyrrolizidine alkaloid, was fed at a dietary concentration of 50/10$^6$ for 55 weeks, to 20 male F-344 rats. Malignant tumours developed in 17/20 animals between 48 and 59 weeks. Forty-five percent (9/20) developed angiosarcomas of the liver and 35% (7/20) had hepatocellular carcinomas. Other tumours included malignant adnexal tumour of the skin (1 rat) and lymphoma (1 rat). Lung metastases were observed in 4 animals with angiosarcoma of the liver and one animal with hepatocellular carcinoma. From one animal, angiosarcoma was successfully transplanted through 4 generations.**

PYRROLIZIDINE alkaloids constitute a large group of naturally occurring hepatotoxins, derived from plants of unrelated botanical families that have a global distribution. These alkaloids are known to cause acute and chronic liver injury in grazing animals. Moreover, these toxins are also implicated in the causation of human liver diseases such as veno-occlusive disease, cirrhosis of liver and possibly hepatocellular carcinoma (Schoental, 1968, 1972). Because of possible contamination of foodstuffs and their use as herbal remedies in developing nations, these alkaloids are considered as health hazards to man.

Chronic administration of some of these alkaloids is known to cause liver tumours in experimental animals (Schoental, Head and Peacock, 1954; Harris and Chen, 1970). However, in most of the earlier studies crude extracts of these alkaloids were used. Using pure crystalline lasiocarpine, an alkaloid derived from *Heliotropium lasiocarpium* or *H. europeum*, Svoboda and Reddy (1972) reported an incidence of 61% hepatocellular carcinomas and 33% squamous cell carcinomas in rats after repeated i.p. injections. The present study deals with the effects of chronic dietary administration of lasiocarpine to rats.

### MATERIALS AND METHODS

Thirty male inbred F-344 rats (Simonson Lab. Inc., Gilroy, California, USA) weighing 80–100 g were housed in individual cages. Pure crystalline lasiocarpine (Chemasea Manufacturing Pty Ltd, Peakhurst, New South Wales, Australia) dissolved in 0·1N HCl was mixed in powdered Purina rat chow (Ralston Purina Co., St Louis, Mo.) at a concentration of 50/10$^6$. The lasiocarpine diet was prepared once in 2 weeks and stored in the refrigerator at 4°C because of the better stability of lasiocarpine at this temperature (Jago, M., personal communication). Twenty rats were fed lasiocarpine diet for 55 weeks. Complete necropsies were performed on all the animals that died or were killed at the end of 59 weeks. Ten control animals were fed rat chow without lasiocarpine. All the control animals were killed at the end of 59 weeks. Tissues from selected organs were processed for light microscopy. Paraffin sections were routinely stained with haematoxylin and eosin, and reticulin stains.

For tumour transplantation, F-344 strain male weanling rats weighing 40–60 g were used. Under sterile conditions, portions of

Correspondence to: Dr M. S. Rao, Department of Pathology, Northwestern University Medical School, 303 East Chicago Avenue, Chicago, Illinois 60611.

TABLE—*Survival, Incidence and Pattern of Tumours in Male F-344 Rats Fed with Lasiocarpine in Diet at a Concentration of 50 parts/$10^6$ for 55 weeks*

| Number of animals started | Treatment | Effective number of animals | Number of animals with tumours | Types of tumours: Angio-sarcoma of liver | Hepato-cellular carcinoma | Others |
|---|---|---|---|---|---|---|
| 20 | Lasiocarpine | 20 | 17 (85%) | 9 (45%) | 7 (35%) | 2 (10%)* |
| 10 | Control | 10 | 0 | 0 | 0 | 0 |

* Others: Malignant lymphoma (1 rat). Malignant adnexal tumour of the skin was observed in 1 rat in addition to angiosarcomas of the liver.

tumour tissue were minced into 1–2 mm sized pieces in normal saline. Two to three of these pieces were placed s.c. in the inguinal region of recipient animals under Metofane anaesthesia. Tumour tissue from the second transplant was processed for electronmicroscopy by conventional methods.

### RESULTS

The survival pattern, tumour incidence and types of tumours observed in rats fed lasiocarpine are summarized in the Table. The earliest tumour was observed in an animal that died during the 48th week. Grossly, the livers contained one or multiple nodules of 1–2·5 cm size. Some of these tumours were brownish and haemorrhagic and others were grey and firm. Histologically, the liver tumours were angiosarcomas in 45% and hepatocellular carcinomas in 35% of the animals. The angiosarcomas contained well-formed angiomatous areas to poorly differentiated spindle-cell regions (Fig. 1). Reticulin stains revealed luminal arrangement of the tumour cells (Fig. 2). The hepatocellular carcinomas were of a well-to-poorly differentiated type. Both these histological types of tumours were not observed together in any animal. Other neoplasms that were noted included malignant lymphoma involving peripancreatic and portahepatic lymph nodes (1 rat) and malignant adnexal tumour of the skin of anterior abdominal wall (1 rat). Metastasis to lungs was observed in 4 animals with

FIG. 1.—Angiosarcoma of the liver showing, angiomatous (well-differentiated) and poorly differentiated areas. H. & E. ×250.

FIG. 2.—Reticulin stain demonstrating the encased endothelial cells within the basal lamina. ×250.

Fig. 3.—Microphotograph of second generation of angiosarcoma transplant. H. & E. ×250.

angiosarcomas and one animal with hepatocellular carcinoma. The histological changes in the non-tumourous areas of the liver included megalocytosis, intranuclear inclusions, hyperplastic nodules, fatty changes, bile-duct proliferation, peliosis hepatis and focal or diffuse hyperplasia of endothelial cells.

The primary transplants from the angiosarcoma grew to a palpable size in 8–12 weeks in the first generation. The subsequent transplants became palpable in 5–8 weeks. Histologically, these transplanted tumours resembled the original tumour (Fig. 3). Electronmicroscopic examination of the second transplant showed neoplastic endothelial cells lining the vascular lumen in well-differentiated

FIG. 4.—Electronmicrograph of malignant endothelial cells lining vascular spaces. ×4000.

areas (Fig. 4). In poorly differentiated areas the tumour cells were spindle-shaped and showed pinocytic activity, cytoplasmic filaments, variable numbers of cytoplasmic organelles and, in an occasional cell, structures resembling Weibel–Palade bodies. These ultrastructural features are very similar to those reported by others (Toth, 1973; Rosai *et al.*, 1976).

### DISCUSSION

It is apparent from these studies that chronic dietary administration of lasiocarpine to rats induced both angiosarcomas and hepatocellular carcinomas in the liver between 48 and 59 weeks. On the basis of food consumption measurements, the average cumulative dose for lasiocarpine was estimated as 190–200 mg. In contrast, repeated i.p. injection of lasiocarpine with a cumulative dose of 125 mg resulted in hepatocellular carcinomas in 61% and squamous cell carcinomas of the skin in 33% of the rats (Svoboda and Reddy, 1972). In an earlier investigation by Harris and Chen (1970) in rats fed *Senecio longilobus*, about 33% developed hepatocellular carcinomas and 4% had angiosarcomas. Proliferation of endothelial cells was also observed in rats administered isatidine in drinking water (Schoental *et al.*, 1954). This difference in histogenetic types of tumours in the liver illustrates the effect of the dose and mode of administration of various alkaloids on the susceptibility of different cell types in a given target organ.

Lasiocarpine has both antimitotic and carcinogenic properties (Jago, 1969; Svoboda and Reddy, 1972). Because of the antimitotic properties it is claimed by Svoboda and Reddy (1972) that treatment of lasiocarpine has to be interrupted for the expression of carcinogenic properties. However, in the present study, different types of tumours were developed even when the animals were on continuous treatment with lasiocarpine. Probably some of the cells escaped the antimitotic action of lasiocarpine, but were susceptible for carcinogenic action. Similarly, development of malignant tumours was also noted in animals concomitantly treated with lasiocarpine and aflatoxin $B_1$ without interruption of treatment (Reddy and Svoboda, 1972).

### REFERENCES

HARRIS, P. N. & CHEN, K. K. (1970) Development of Hepatic Tumors in Rats Following Ingestion of *Senecio longilobus*. *Cancer Res.*, **30**, 2881.

JAGO, M. V. (1969) The Development of Hepatic Megalocytosis of Chronic Pyrrolizidine Alkaloid Poisoning. *Am. J. Pathol.*, **56**, 405.

REDDY, J. K. & SVOBODA, D. J. (1972) Effect of Lasiocarpine on Aflatoxin $B_1$ Carcinogenicity in Rat Liver. *Arch. Pathol.*, **93**, 55.

ROSAI, J., SUMNER, H., KOSTIANOVSKY, M. & PEREZ-MESA, C. (1976) Angiosarcoma of the Skin. *Human Pathol.*, **7**, 83.

SCHOENTAL, R., HEAD, M. & PEACOCK, P. (1954) Senecio Alkaloids: Primary Liver Tumors in Rats as a Result of Treatment with (1) a Mixture of Alkaloids from *S. jacobaea* Lin, (2) *Retrorsine*, (3) *Isatidine*. *Br. J. Cancer*, **8**, 458.

SCHOENTAL, R. (1968) Toxicology and Carcinogenic Action of Pyrrolizidine Alkaloids. *Cancer Res.*, **28**, 2237.

SCHOENTAL, R. (1972) Herbal Medicines to Avoid. *Nature, Lond.*, **238**, 106.

SVOBODA, D. J. & REDDY, J. K. (1972) Malignant Tumors in Rats Given Lasiocarpine. *Cancer Res.*, **32**, 908.

TOTH, B. (1973) 1,1-Dimethylhydrazine (Unsymmetrical) Carcinogenesis in Mice. Light Microscopic and Ultrastructural Studies on Neoplastic Blood Vessels. *J. natn. Cancer Inst.*, **50**, 181.